# An immersive mirror: a descriptive study of peer observer and active participant experiences in simulation

**DOI:** 10.1186/s41077-025-00395-7

**Published:** 2025-12-02

**Authors:** Naomi Tutticci, Sandra Johnston, Joanne Ramsbotham, Karen Theobald

**Affiliations:** 1 School of Nursing and Midwifery, Griffith University, 177 Kessels Rd, Nathan Qld, Brisbane, 4111 Australia; 2 School of Nursing, Queensland University of Technology, Brisbane, Australia

**Keywords:** Peer observer, Simulation, Nursing, Pre-registration, Empathy, Holistic

## Abstract

**Background:**

There is limited evidence and humanistic thinking about the thoughts and reactions of peer observers during nursing simulation. An increased understanding may provide new insights and opportunities to advance therapeutic relationships and holistic care. This study explored peer observer and active participant thoughts during simulation to better understand how shared learning experiences transform and improve nursing practice.

**Methods:**

A qualitive descriptive design generated data via peer observers and active participants’ self-reported experiences from pre-registration second-year, nursing students. Responses were synthesized and analyzed using reflexive thematic analysis.

**Results:**

From 175 peer-observer accounts, four codes were generated and synthesized into three themes: Observer self-critique and critique of others; observer empathy and affect; and observers’ outsider perspective. Six codes were generated from the analysis of 234 active participant accounts analysis and synthesized into three themes: participant affect; participant cognition and participant confidence.

**Conclusions:**

The peer observer role can experience simulation as an immersive and emotive encounter that may indicate active and deep learning is occurring. Simulation learning design should prioritize the identification of empathy experienced by observers for the participants and explicitly include it in cognitive processing undertaken during simulation debrief. Linking the experience of empathy with nursing theory in simulation is a powerful learning tool.

**Supplementary Information:**

The online version contains supplementary material available at 10.1186/s41077-025-00395-7.

## Introduction

Many nursing students have limited experience caring for, and an understanding of patients lived experiences. As faculty, we need to be mindful of purposefully balancing learning in the intuitive art, scientific knowledge, and technical capability domains of nursing when preparing undergraduate nursing students [1]. Simulation provides a safe, reality-based and interactive environment for students to practice their abilities, make mistakes and repeat the process leading to mastery [2]. – [3] This provides an opportunity for students’ active participation and direct monitoring by their peers, improving their understanding of care provision because it is seen and applied [3]. Holistic nursing simulation combines simulation pedagogy with holistic nursing philosophy promoting students’ self-awareness and a grasp of others’ perspectives in a caring and healing relationship [4]. 

The active participant experience in simulation has been extensively evaluated and is largely understood with recent refinements to simulation design evolving as experiential learning for both participant and observer is prioritized. The active participant and observer simulation model is commonly used, allowing participants to immerse themselves safely, in real-world clinical scenarios [5]. In this simulation model, observers typically provide feedback during debriefing with facilitator guidance [6]. To date, simulation design and implementation have focused on optimizing this real-world, simulated experience. This is not disputed as being of value as it is essential for improving the student learning experience. Little is known however about peer observer thoughts during simulation and further how an understanding of this phenomenon may translate to improvements in simulation design and implementation [2]. Peer observers learn vicariously in simulation-based experiences (SBE) but also may experience similar thoughts and reactions to active participants. This paper argues that peer observers could experience both vicarious and immersive learning, a position not universally evident in contemporary literature [7]. Similar thoughts and reactions by both peer observers and active participants suggest an emerging simulation phenomenon, worthy of further exploration if such an approach optimizes understanding of each other as whole beings [4]. 

## Background

Simulation can incorporate both an ‘active participant’ and ‘peer observer role’, the active participant is required to immerse themselves in an allocated role, in the simulation scenario, whilst the observer is typically physically distanced from the scenario, whilst documenting observations via a worksheet or cognitive aid that focusses attention and scaffolds thinking [5, 8]. While the participant experience of simulation has been well documented, what is less understood is peer observer reactions during simulation.

Observation of simulation aligns with vicarious learning principles. Vicarious and immersive learning have appeal for the adult learner, with benefits attributed to both forms of learning [9]. Vicarious learning or learning from the behavior of others is a concept derived from the work of Bandura [10]. Watching your peers in the simulation observer role can provoke learning through an alternate mode and reduce the cognitive load associated with immersive learning, particularly when new skills are being acquired [11]. Numerous advantages are associated with the peer observer role, including learning from an objective position, opportunities for reflective practice and increasing student participation in the experience [12]. Conversely, observation as learning is not considered to be a true form of experiential learning as active participation is required [12]. Conceptually, vicarious learning via observation of peers during simulation has been posed as more valuable than active participation especially when combined with evaluative peer feedback [13]. Learning through the observation of others is a known concept but it has not been investigated thoroughly as an aspect of experiential learning in simulation contexts [12, 14]. 

Understanding how quality SBE learning outcomes are obtained is obscured when the peer observer role is poorly reported and studies rarely distinguish between active participant and observer [5, 7] as this limits a deeper conceptual understanding of the shared functionality of the roles and the distinct differences. To optimize learning when physically separated whilst maintaining an intellectual connection with the scenario, a structured approach to peer observation is often used, such as a worksheet [8]. If faculty value, the role of observer as critical for the learning potential associated then this role may be fully realized [9]. 

This study aimed to explore and describe peer observer and active participant thinking during simulation, to better understand shared learning experiences. Analysis of written responses to the question: ‘What were you thinking’ during a simulation-based experience (SBE) aimed to provide insight and increased clarity of student thinking, and to inform faculty and students about the impact of holistic nursing simulation.

Examination of the impact of vicarious and active learning on student learning in this study was underpinned by social constructivism [15] and Kolb’s experiential learning framework,^16^ which emphasizes the importance of social connectedness in learning interactions. Kolb’s work encourages learners to link their ‘new’ knowledge and experience to existing, thereby assisting them to broaden world views and challenge assumptions [16]. In this way experiential learning assists learners in making sense of their learning and integrating their knowledge in a wide variety of contexts. The guiding theory of social constructivism and framework of experiential learning were the lenses that researchers used to guide the research process.

## Methods

This study used a qualitative descriptive design to capture and explore nursing students’ thinking whilst in the role of simulation peer observer and active participant, after having engaged in a simulation-based experience *(SBE*). An open-ended question was posed to all nursing students post SBE via an online survey. The study was conducted following the O’Briens’ et al. standards for reporting qualitative research [17]. 

### Setting and sample

The setting for the study was an Australian university with a large Bachelor of Nursing second-year nursing clinical subject, comprised of preparatory theoretical content for laboratories and clinical placement. Students attended one 2-hour facilitated simulation which focused on a deteriorating patient scenario. A convenience sample of students enrolled in the second-year clinical subject (*n* = 1000) were eligible for inclusion in the study, there were no exclusion criteria. SBE facilitators delivered pre-brief to orientate students to the simulation objectives and role of participant and observer. Debriefing of both participants and observers immediately followed the simulation.

### Data collection

Qualitative data was generated via peer observers and active participants’ self-reported experiences after participating in an SBE. Demographic information collected from participants included age, gender, enrolment status (part-time/full-time), the number of previous simulation sessions attended, course enrolment (degree, accelerated entry), and if previous tertiary study had been undertaken before the current course enrolment. All participants were asked to indicate if they were an observer or active participant.

Participants were surveyed post-simulation using Key Survey, a web-based survey platform that asked an open-ended question depending on their role as either observer/active participant: ‘*What were some of the thoughts going through your mind when you were observing others’ practice?*’ or ‘*What were some of the thoughts going through your mind when you were actively participating in the simulation?’*

### Procedure

After ethical approval, potential participants were advised of the study through electronic announcements and emails distributed through the university’s learning management system. At the university where this study was undertaken, a simulated learning experience was a standard element of their enrolled subject. Students self-allocated to a simulation group according to their availability. Class size was capped at eight students per simulation class and there were 129 simulation sessions in total.

On the day of their scheduled simulation class, a research assistant invited students to participate in the study. Participant Information Sheets were provided and informed the participant of the study aim, procedure, and associated risks. Participants could opt out of the study at any time without penalty. At the commencement of the simulation class, students self-selected to be an active ‘participant’ (4 students) in the simulation or a ‘peer observer’ (4 students). The simulation facilitator delivered an orientation to the simulation room and a pre-brief which included addressing three broad domains of learning – communication: patient assessment and teamwork (10 min). The students who were active participants then participated in a 20-minute clinical scenario using 3G SimMan™. The scenario was about a 55-yr old man who had undergone a percutaneous coronary intervention and subsequently developed chest pain, whilst in recovery. During the simulation participants were expected to demonstrate patient assessment, management of the patient’s chest pain and effective communication with the patient and other members of the healthcare team. The participants who were peer observers sat in an observation room and completed an observation form < Supplementary File>. After the debriefing, participants were asked to complete a web-based survey, accessed via a QR Code or survey link. Completion of the survey indicated informed consent.

### Data analysis

Peer observers’ and active participants’ accounts of thinking were synthesized and analyzed using thematic analysis [18]. Initial and subsequent codes were identified using an iterative approach. They were questioned and reviewed by the researchers independently and then together, to ensure no assumptions were applied and the focus was on keeping true to the research aim [19]. Similar codes were color-coded, collated and refined to generate themes, reflective of our interpretation of stories about the data under investigation [19]. Our process was collaborative and reflexive, staying true to the data and relationships, and holding true to our theoretical understandings underpinning this research [20]. The researchers engaged in personal, professional and contextual reflexivity during the study as they were motivated to understand student simulation experiences. The intent was to inform teaching practices and to reshape and improve simulation design and delivery [21]. Interpersonal reflexivity was required, as a power differential existed between researchers (teachers) and participants (students) in this study. Participants were informed, during the consent process, that study participation was optional and would not impact on grades. Regular collaborative efforts were undertaken by the research team to ensure that existing intra-professional power dynamics did not interfere with data analysis.

## Findings

Most of the participants were female (78%), between 21 and 30 years (53.8%), and enrolled full-time in the undergraduate Bachelor of Nursing program (97.1%). Of the four entry options to the BN program, the majority were Baccalaureate (previous degree) (35.8%) who had one previous simulation experience (49.2%) (Table 1). There were one hundred and seventy-five (*n* = 175) peer observer accounts and *n* = 234 active participant accounts analyzed.

**Table 1:** Participant characteristics

Characteristics	Number	Percentage
**Age**,** mean**,** SD**	25.6 (6.8)	
**Age groups**		
< 20 years	107	25.9%
21–30 years	222	53.8%
31 and above	77	18.6%
Missing	7	1.7%
**Gender**		
Male	87	21.1%
Female	322	78%
Not specify	4	0.7%
**Enrolment**		
Full time	401	97.1%
Part time	12	2.9%
**Previous simulation sessions**		
0	160	38.7%
1	203	49.2%
More than 1 (from 2–20)	42	10.1%
Missing	8	1.9%
**Course enrolment**		
Three-year degree	118	28.6%
Baccalaureate (prior degree)	148	35.8%
Diploma entry (prior diploma)	81	19.6%
Double degree nursing/other discipline	56	13.6%
Missing	10	2.4%
**Previous tertiary study before this course**		
No, first experience	155	37.5%
Yes, vocational study	97	23.5%
Yes, other university study	158	38.3%
Missing	3	0.7%
**Observer or not**		
Yes	175	42.4%
No	234	56.7%
Missing	4	1.0%

### Themes (Peer observer)

From the 175-peer observer accounts, four final codes were identified. An iterative process was applied to collapse initial to final codes. For example, similar initial codes such as ‘questioning self’, ‘questioning others, and self-doubt’ were combined into one code of ‘critique of self and peers’, the final four codes were synthesised by researchers (initials removed for review) into three themes (our analytical interpretations): Observer self-critique and critique of others; observer empathy and affect and observers’ outsider perspective (Table 2). Participant’s accounts may have fallen into more than one area of data analysis.

**Table 2:** Codes, theme, and frequency of peer observer thinking organized by analytical interpretation

Code: Critique of self and peers n = 104 Theme: Observer self-critique and critique of active participant	Codes: Being empathetic n = 39 and Emotional response n = 35 Theme: Observer empathy and affect	Code: Distance from the simulation n = 26 Theme: Observers’ outsider perspective

### Observer self-critique and critique of others (active participant)

Observer self-critique and critique of others was the dominant theme from the observer perspective (*n* = 104), with variation in critique from descriptive #51 *The [participant] students did very well”* to higher level evaluation #176 “*We had an excellent team leader who guided the simulation. They were aware of what needed to be done regarding patient assessments. Their assessments could have been more thorough and precise however.*

Minimal observer comments (10.6% or *n* = 11) included both self and critique of an active participant. Participant *#78* best describes this dual observer role as both self and other critique when they commented *I considered what I would’ve done differently but also recognized that within the moment with the intensity and subconscious stress placed upon the students*,* I likely would’ve responded similarly. I also could recognize how easily students can forget fundamental skills when under pressure.*

### Observer empathy and affect

Peer observer empathy and affect demonstrated that an emotional response was elicited by observers watching active peers participating in the simulation.

#97 *It was frantic and I was glad I wasn’t a participant. Everyone was obviously nervous and didn’t know one another.*

An emotional connection with the active participants was evident with empathetic accounts given by observers, highlighting a willingness to vicariously connect themselves to their peers and provide them with support.

*#112 That I wanted to help the participants. They were stressed which made me feel stressed. I was also trying to put myself in their roles and wonder if I would be able to handle the stress better or not.*

Not only did observers articulate an emotional connection with the active participants, but they desired to assume the role, whilst watching to improve how they could cognitively engage with the scenario. Participant #123 wanted to *…put myself as a participant*,* thinking what should I do if I dealt [sic] with this patient.*

### Observers’ outsider perspective

The ‘*observer as an outsider’* perspective typically identified observation as an easier role to assume in the simulation experience, with a perception of ‘distance’ between observer and active participant enabling a clearer view of what behaviors were required, completed, or omitted.

#8 *It was a lot easier seeing the simulation from an outsider’s perspective. All observers commented on things they did do*,* didn’t do and should have done* and participant #108 stated *It was easier to see from a distance some of the key things the participants were missing.*

Despite categorisation of accounts into three themes, participant accounts also indicated observer capacity to simultaneously be objectively critical of self and their peers, yet emotionally and empathetically connected.

### Themes (Active participants)

From the 234 active participant accounts, six final codes were identified. An iterative process was applied to collapse initial to final codes. For example, similar codes such as ‘blank mind’, ‘brain freeze’ and ‘hard to problem solve’ were combined into one code ‘blank mind’. These final codes were reviewed, refined, and synthesized by researchers (initials removed for review) into three themes, our analytical interpretations: participant affect; participant cognition and participant confidence (Table 3).

**Table 3:** Codes, themes and frequency of active participant thoughts organised by analytical interpretation

Code: Emotion (n = 98) Theme: Participant Affect	Codes: Applying theory to practice n = 50; blank mind n = 11 and time n = 11 Theme: Participant Cognition	Codes: Self-doubt n = 20 and self-improvement, self-critique n = 42 Theme: Participant Confidence

### Participant affect

Participant affect was the dominant theme generated. Participant affect encompassed a continuum of emotion from panic (negative) to excitement (emerging positive), including in the words of one participant #72 *“pure terror’*, although the predominant cluster of emotions were panic, stress, nervousness and feeling overwhelmed. When excitement was mentioned, it was less frequent and usually in the context of an initial feeling of nervousness or combined nervousness with excitement.

### Participant cognition

Participant cognition likewise was interpreted as a continuum of conscious thought from an empty mind to clear, intentional thoughts about how to apply what was learnt in class to the simulation scenario, as the patient was deteriorating. Participant #89 captures the thrust of altered cognition when engaged in simulation with this statement, *When the emergency came out*,* the knowledge and theories that I have learned could not be performed. Instead*,* my mind just went blank.*

When participants mentioned time, it featured as a block to thinking and meant there was insufficient time to respond to the simulation scenario as experienced by active participant #192’…*It all happened so quickly and it’s not that I forgot what I had learnt but in feeling flustered just didn’t take the time to properly process things. I also got distracted at points thinking about pathophysiology which was fun intellectually but slowed me down.*

### Participant confidence

Participant confidence ranged from self-doubt about the capacity to problem solve under simulation conditions, to self-critique whilst in an active state (thinking and doing). Some students suggested how they could improve their simulation experience based on being able to observe their active peers and their performance. Self-critique included critique of team performance from the perspective of the participant as well as the individuals’ performance.

### Active participant: the relationship between affect, confidence, and cognition

There was a cross-over noted between two of the three themes- analytical interpretations for active participant. These crossovers were between: Participant Affect and Participant confidence and Participant affect and Participant cognition. There was no noted cross-over of themes within the Peer Observers.

The relationship between active participant affect and confidence was characterised by heightened negative emotion (panic and nervousness) and expressive self-doubt. Active participant *# 269* conveyed stress and self-doubt, highlighting their uncertainty to continue with the simulation experience, even when simulation pre-learning had been completed.

*I felt very nervous and unsure of myself even having completed all pre-learning materials.*

Similarly, participant affect impacted on capacity to think clearly or even begin to process information arising from the simulation. As emotion intensified, within a grouping of nervousness, stress and panic, active participants recounted a tendency for what may be interpreted as harried thinking. Participant #121 identified the value of having previous real-life experience in an emergency, as a tool, to mitigate the impact of strong emotions on their thinking during the simulation.

*A lot of thoughts going through my mind during the simulation. I have the knowledge but it’s hard for me to apply it due to mixed feelings of anxiousness and nervousness. Overall*,* I learned a lot from this simulation. Helped me to have a little bit of background in a real-life emergency situation*.

Only two of the active participants (0.9%) relayed strategic thinking during simulation. These participants were intentional in applying theory to practice yet also had emotional accounts like others. One of the two participants, *#233*, articulated an initial emotional response, followed by a conscious cognitive action - *‘I talked to* myself’ and then they focused thinking on applying what was learnt from the Clinical Practice Sessions (CPS) (clinical skill laboratories).

*Firstly*,* I felt nervous then I talked to myself: and then conducted what I learned from CPS class.*

Active participant #233 acknowledged their emotion early when engaged in a potentially challenging learning activity and then consciously focused their thinking on the target area of learning to be acquired. Calling on previous understandings combined with emotional awareness seemed to facilitate application. The only other combined response of participant affect and cognition (applying theory to practice) was by participant # 234

*I was nervous but excited. During the simulation many assessments came to my mind to perform on the patient*,* and I did not know where to start*,* but our group had good communication so we worked it [sic] out.*

Successful simulation learning for this participant was moderated by emotional accounts and quality emotional processing, brokered by the team members.

Conversely, only 20.5% of active participants reported their thinking focused solely on the application of theory to practice with no emotional component.

### Combined analysis of peer observer and active participant thinking

Affective accounts about simulation were a shared experience regardless of the role occupied by second-year nursing students during simulation. Whilst the active participant was experiencing a continuum of emotion from nervousness to panic, the observer was vicariously experiencing a similar emotional/stress continuum, with the addition of empathy for the active participant. Empathy for patients is well-recognized as a key attribute of nursing practice and person-centred care [22]. Critique of self was jointly experienced by peer observers and active participants, suggesting simulation is an effective tool for reflective practice which can be promoted within debriefing.

## Discussion

The emotional response elicited by simulation experience is well documented, especially about patient care [23]. This study used a simulation case with a deteriorating patient case scenario, so emotive responses were expected. Two interesting results arose from this study: (1) observers empathizing with their peers (active participants) with the experienced emotion (empathy) driving learning and (2) a shared mindset and parallel feelings of peer observers and active participants. Peer observers’ emotional and cognitive responses about the active participants indicate that SBE can elicit empathy between peers. The significance of shared experiences between peer observers and active participants in simulated learning, prompts possible re-evaluation of current debriefing practices. Currently, the two roles are considered as discrete experiences and are reported as such in the literature, with standards for simulation design omitting the observer role [24]. This research confirms the value of including peer observers in the simulation experience [5] by capturing peer observer thinking as astute yet al.so aligning with a range of emotional accounts of the active participant. The value of the observer as an empathetic, evaluator of simulation is a desirable professional attribute [25]. 

This study recommends educators extend the scope of debriefing to include explicit identification of empathetic accounts from observers. A discrete learning outcome, such as ‘identify and describe empathetic thinking and behaviors’ can assist with the development of therapeutic, person-centred and professional relationships.

Educators cannot underestimate the value of students experiencing empathy for patient groups in a learning situation, particularly in a relatively controlled experience of simulation [22, 23]. Simulation debriefing has evolved to recognise emotional processing for active participants and observers by allowing time at the commencement of debriefing for this to take place [26]. Under-represented within the current simulation and debriefing approaches is reflection on empathy, as experienced by active participants and observers. Educators can acknowledge empathy, whether it spontaneously occurs when peer feedback is provided or is facilitator prompted. In this way students in simulation are connecting in a community of practice through their shared experiences and then can connect this emotion to their clinical practice. This significant finding supports explicit teaching of this ‘soft skill’ which is critical for the effective delivery of person-centered care [22] along with teamwork between peers.

Simulation, inclusive of peer observers can build the capacity to transfer empathetic interactions during clinical care by undergraduate students. Debriefing needs to occupy a central role in building this shared connection between observer and participant, reflecting on how it feels to be empathetic and what reaction it elicits (behavioral). Empathy is not universally intuitive and explicit teaching and modelling, as for reflection, needs to occur. [27, 28] Similarly learning from strong emotions may be prompted. Ongoing research is required to better understand how empathy-based care can be optimized in simulation as a legitimate mode of learning.

Students readily identified with their peers when learning was challenging. Simple clinical intention, skill and actions were forgotten when pressure was present. Nuances of the peer observer role require attention to improve vicarious learning and post-simulation quality reflection. The intense emotions and stress response experienced when observing your peers can limit cognitive processing. The authors recommend adjusting debriefing practice to include emotional processing at the commencement of debriefing, for peer observers along with active participants. Learning within cognitive processing post-simulation has been shown to improve when the emotional response to the simulation experience has been elicited, acknowledged and processed [26]. 

A cognitive load has been demonstrated by both peer observers and active participants, which can limit deeper and sustained learning. Whilst this is not a new finding, it suggests that in simulation contexts underestimation of the intensity of immersion continues. The importance of empathy-based learning within a simulation experience can expand the application of simulation as a pedagogy in preparing nursing students for person-centered and holistic care. The opportunity to experience ‘empathic humility’ is shared within a tribe (sic.) of nursing students [29] immersed in simulation.

### Limitation of study

Qualitative data of peer observer and active participant’s thinking was a snapshot from a moment in time, post-simulation completion. Data collected at one time point, is limited in establishing relationships or cause and effect. Participant translation of thinking to practice was not captured for analysis. Peer-to-peer empathy and the role empathy played in cognitive processing were the main findings of this study and although the authors pose that this is a transferable skill, this was not explored, limiting inference to application to clinical practice.

## Conclusion

In summary, the analysis of peer observer and active participant thoughts and reactions captured in this study provided unique insights into students’ experiences of delivering therapeutic care within a simulated environment. In planning simulation experiences faculty should activate the observer role as an opportunity for prioritizing the art (empathy) and science (cognitive processing of best practice) of holistic nursing care. Increased awareness of empathetic practice can be achieved by adopting a stop/micro-debrief/restart during simulation, which can include specific prompts by facilitator to link expressed empathetic thoughts to clinical practice. Similarly, observer worksheets can include prompts to document empathetic thoughts towards their active participant peers. In this way, the observer role and associated activities are scaffolded to promote active learning over sustaining a passive approach which can limit dynamic interpersonal interactions. Such approaches will maximize exposure to critical elements of holistic nursing, such as empathy-informed care. This study has highlighted how those in the peer observer role can experience simulation as an immersive and emotive experience that may indicate active and deep learning is occurring. As faculty, we can improve nursing students’ capacity to apply empathy in practice by creating simulations that harness/target the experience of empathy (art) alongside the science of therapeutic care (cognition processing).

Additionally, if such experiences mirror the learning active participants achieve and if this enables later transfer of learning to real care contexts, it is an area for future investigation.

## Supplementary Information

Supplementary Material 1.

## Data Availability

The datasets used and/or analysed during the current study are available from the corresponding author on reasonable request.
