# Supplementary material for: An immersive mirror: a descriptive study of peer observer and active participant experiences in simulation
Source: Adv Simul (Lond). 2025 Dec 2;11:1. doi: 10.1186/s41077-025-00395-7 (PMC12777330; doi:10.1186/s41077-025-00395-7)

**CRiticAL – clinical reasoning observer worksheet (CROW)**

**Directions:** Please make notes on the whole episode of care using the clinical reasoning cycle. Focus on appraisal of participants’ practice observed, **what you would have done, what went well**, note anything **missed or overlooked and points for improvement**. You will use these notes to provide feedback during the debrief.

| **Key learning points in the scenario**   - Identify principles for assessment and management of clinical and supportive care needs. - Identify strategies that can facilitate/impede effective communication in the context of an individual’s response to loss and grief, situational uncertainty (including uncertainty about illness trajectory) and changing goals of care. - Identify factors that can facilitate/impede effective team work between members to maintain quality of life for patients with a life limiting illness, their families and care givers. | |
| --- | --- |
|  | |
| **Clinical reasoning cycle** | **How did the simulation participants address the key points in the scenario?**  **Make notes below** |
| - **Consider person & context** - **Collect cues/ information** - **Process information** | **Assess**  What data did participants collect? Was it holistic?  How was data collected? Suitable framework used?  Any recommendations for further assessment data collection? |
| - **Identify problems / issues** - **Establish goals** | **Plan**  What are normal/abnormal findings from data collected?  What do you think the patient problems are?  What are your priorities for action? Same as observed? Why? |
| - **Take action / intervene** | **Implement**  What interventions did the participants implement?  Were the interventions relevant to the scenario?  Do you agree with interventions undertaken?  What other interventions could have been done? Provide a rationale? |
| - **Evaluate outcomes** - **Reflect on process & new learning** | **Evaluate**  How did the patient’s condition change?  Were the interventions effective? How did you know this?  What could be improved in future similar situations?  Any recommendations to improve future team communication? |

**Clinical Reasoning Cycle**


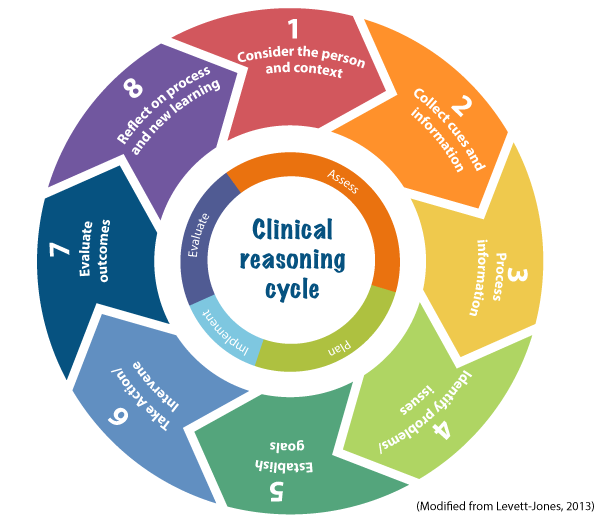

Supplement: Supplementary file 1 — Supplementary Material 1. [file 41077_2025_395_MOESM1_ESM.docx]
